# A Synthetic Peptide Shows Retro- and Anterograde Neuronal Transport before Disrupting the Chemosensation of Plant-Pathogenic Nematodes

**DOI:** 10.1371/journal.pone.0017475

**Published:** 2011-03-07

**Authors:** Dong Wang, Laura M. Jones, Peter E. Urwin, Howard J. Atkinson

**Affiliations:** Centre for Plant Science, University of Leeds, Leeds, United Kingdom; Ghent University, Belgium

## Abstract

Cyst nematodes are a group of plant pathogens each with a defined host range that cause major losses to crops including potato, soybean and sugar beet. The infective mobile stage hatches from dormant eggs and moves a short distance through the soil to plant roots, which it then invades. A novel strategy for control has recently been proposed in which the plant is able to secrete a peptide which disorientates the infective stage and prevents invasion of the pathogen. This study provides indirect evidence to support the mechanism by which one such peptide disrupts chemosensory function in nematodes. The peptide is a disulphide-constrained 7-mer with the amino acid sequence CTTMHPRLC that binds to nicotinic acetylcholine receptors. A fluorescently tagged version of this peptide with both epifluorescent and confocal microscopy was used to demonstrate that retrograde transport occurs from an aqueous environment along bare-ending primary cilia of chemoreceptive sensilla. The peptide is transported to the cell bodies of these neurons and on to a limited number of other neurons to which they connect. It appears to be localised in both neuronal processes and organelles adjacent to nuclei of some neurons suggesting it could be transported through the Golgi apparatus. The peptide takes 2.5 h to reach the neuronal cell bodies. Comparative studies established that similar but less abundant uptake occurs for *Caenorhabditis elegans* along its well studied dye-filling chemoreceptive neurons. Incubation in peptide solution or root-exudate from transgenic plants that secrete the peptide disrupted normal orientation of infective cyst nematodes to host root diffusate. The peptide probably undergoes transport along the dye-filling non-cholinergic chemoreceptive neurons to their synapses where it is taken up by the interneurons to which they connect. Coordinated responses to chemoreception are disrupted when the sub-set of cholinergic interneurons secrete the peptide at synapses that have post-synaptic nicotinic acetylcholine receptors.

## Introduction

Plant parasitic nematodes are major pathogens of plants that cause annual total cost of over $125 b to world crops [1] and are difficult to control. Strategies that focus on the prevention of nematode invasion offer high potential as they prevent the associated mechanical damage in addition to preventing the development of pathogenesis. One strategy to achieve this is to interfere with the orientation of nematodes to roots. They rely on several different gradients some of which attract nematodes to the rhizosphere before others cue orientation to the root and the particular location where invasion occurs. On agar plates the invasive second stage juveniles (J2) of *Heterodera* cyst nematodes aggregate in response to localised 50 mM CaCl_2_ [2] and to root exudates [3] and also orientate to the latter [4]. The nematicide aldicarb, a potent acetylcholinesterase inhibitor, eliminates this aggregation in the locality of CaCl_2_ at about 1 pM, some 10^6^-fold lower than required to affect locomotion [2]. The specific disruption of chemoreception of *Heterodera* and a second cyst nematode (*Globodera pallida*) was also achieved by a peptide mimetic of aldicarb obtained by phage display. It binds to and inhibits acetylcholinesterase, and can be displaced from the enzyme by aldicarb. Inhibition of nematode chemoreception occurred at a peptide concentration that was at least 10^3^-fold less than required to inhibit locomotion [2]. Furthermore a second chemoreception-disrupting peptide (nAChRbp) was obtained by biopanning against a membrane fraction of *Caenorhabditis elegans* that was rich in nicotinic acetylcholine receptors (nAChR). The peptide is displaced from this binding by the anthelminthic levamisole. This second peptide is a disulphide-constrained 7-mer with the amino sequence CTTMHPRLC [2]. The ability of the two peptides to disrupt chemoreception is of interest as both aldicarb and levamisole target cholinergic synapses by interfering with, respectively, enzymatic cleavage of the neurotransmitter acetylcholine and nicotinic acetylcholine receptors. Approximately 115 of 302 neurons are cholinergic in *C. elegans* [5] and the nAChRs associated with them are of particular interest. The nAChRs are ligand-gated ion channels that mediate the action of acetylcholine at neuromuscular junctions and between neurons. Each comprises five subunits arranged in a transmembrane ring with the subunits being designated as either α or non-α type. Individual receptors may be either homomeric or heteromeric. *Caenorhabditis elegans* has the largest and most diverse nAChR gene family characterised to date with 29 subunits assigned by sequence similarity to five distinct groups, two of which are nematode-specific [6]. The five genes *unc-29*, *unc-38*, *unc-63, lev-1* and *lev-8* encode nAChR subunits involved in formation of levamisole-sensitive receptors [7] with a further three ancillary proteins (RIC-3, UNC-50 and UNC-74) required for complete reconstruction of the receptor [8]. The interest in nAChR target screening to develop new veterinary and pesticide products [9] parallels our success in reducing cyst nematode invasion of plants by expressing the levamisole mimetic peptide in their transgenic roots [10].

The aim of the current work is to define how low concentrations of the levamisole-mimetic peptide disrupt chemoreception of cyst nematodes. One clue is that some chemosensory neurons located in the anterior head region of the nematode i.e. the amphidial chemoreceptive neurons, dye fill in *C. elegans* [11], animal parasitic nematodes [12] and cyst nematodes [2]. We now investigate if the peptide enters by this route to reach cholinergic synapses. The high level of conservation of nematode nervous systems facilitates investigation into the neurobiology of cyst nematodes, for which there is limited knowledge. For instance the amphidial neurons of *C. elegans* and those of a soil nematode *Acrobeles complexus* are broadly similar in number and arrangement of cells although these nematodes are in different clades with the latter being a relative of cyst nematodes [13]. Further evidence of similarity comes from heterologous expression studies. *C. elegans* transformed with a GFP reporter construct under control of the promoter of a homologous acetylcholinesterase gene (*ace-2*) from *G. pallida* expresses GFP as expected in the inner labial papillae. The coding region of the *G. pallida* gene also rescues the double *ace-1*, *ace-2* mutant of *C. elegans* to a normal phenotype [14].

We show, using a fluorescently labelled form of the peptide, that it follows the dye-filling route to neuronal cell bodies in the region of the lateral and ventral ganglia. Its ability to disrupt orientation to root exudates does not occur until it has accumulated there in a process that includes slow retrograde transport along dendrites. *C. elegans* also takes up this peptide along its dye-filling neurons to the region of its lateral ganglia but to a lesser extent than the cyst nematodes. We suggest the peptide is delivered by the dye-filling chemosensory neurons to one or more cholinergic interneurons to which they connect. This very specific delivery disrupts the role of the interneurons in chemoreception processes important for normal invasion of plant roots by cyst nematodes.

## Results

### Neuronal uptake of tagged nAChRbp by plant-parasitic nematodes

**Figure 1:**
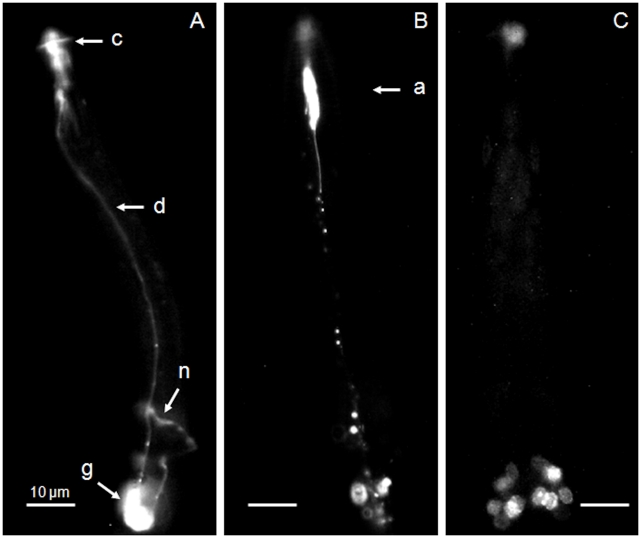
Uptake of fluorescent dyes by neurons of *Heterodera schachtii.* After 16 h exposure to 1 mM of A) FITC B) Alexa Fluor 488 and C) bisbenzimide fluorescence was observed in J2 of *H. schachtii* using epillumination excitation under standard conditions. Images A) and B) are lateral views and C) a dorsal view. Key: c, cephalic framework; (showing autofluoresence); a, amphidial pouch; d, tract of amphidial dendrites; n, nerve ring; g, region of the lateral and ventral ganglia. Scale bars are 10 µm.

**Figure 2:**
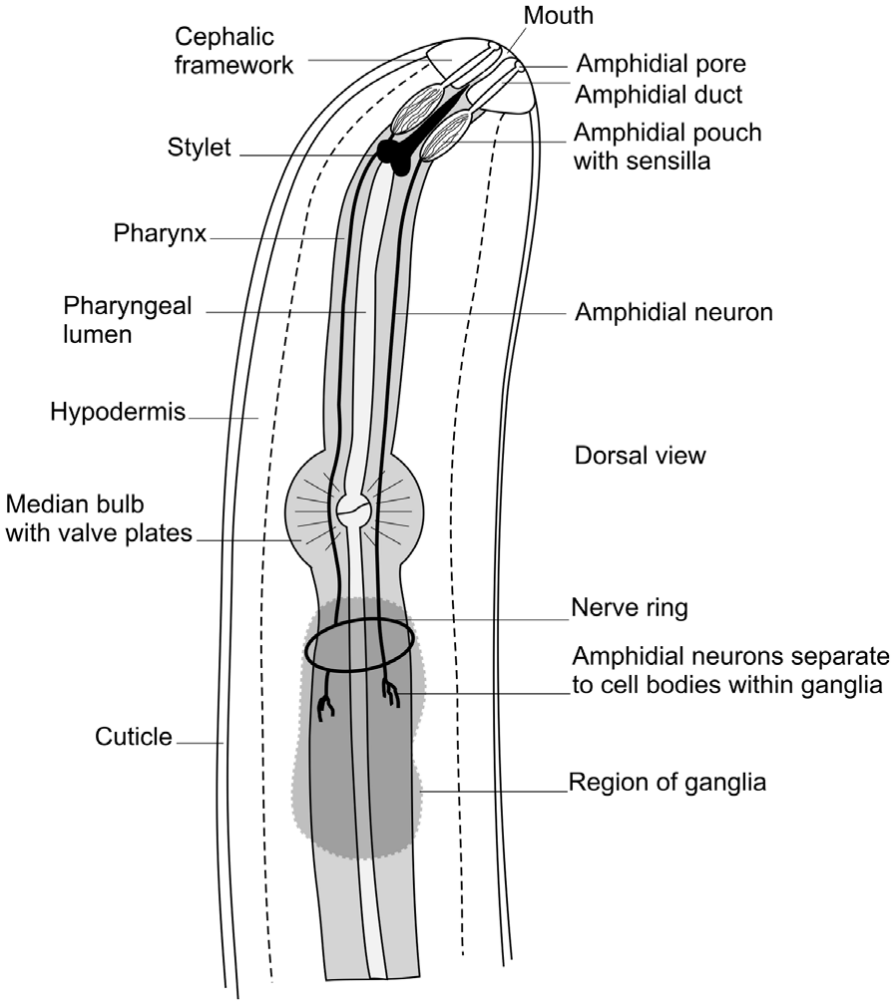
Schematic representation of the anterior region of a cyst nematode. The representation highlights the amphidial structures and neurons and omits oesophageal glands for clarity.

Exposure of J2 *Heterodera schachtii* to 1 mM fluorescein isothiocyanate (FITC) for 16 h resulted in dye-filling of certain amphidial neurons as in previous work [2]. The fluorescence of FITC is evident from the region of the amphids along the tract of at least some amphidial neuron dendrites to their cell bodies and then forward along their short axons to the nerve ring (Fig. 1a). A similar exposure of the nematode to 1 mM Alexa Fluor 488 resulted in dye uptake but with a somewhat different pattern. The region of the amphidial pouch (shown in the schematic diagram Fig. 2) emitted considerable fluorescence but this was much less evident in the region where the cell bodies of the amphidial neurons occur (Fig. 1b). The nuclear staining achieved by 1 mM bisbenzimide was not evident at the amphidial pouches but dye-filling resulted in several nuclei in the region of the amphidial cell neurons being visualised (Fig. 1c) as before [2].

**Figure 3:**
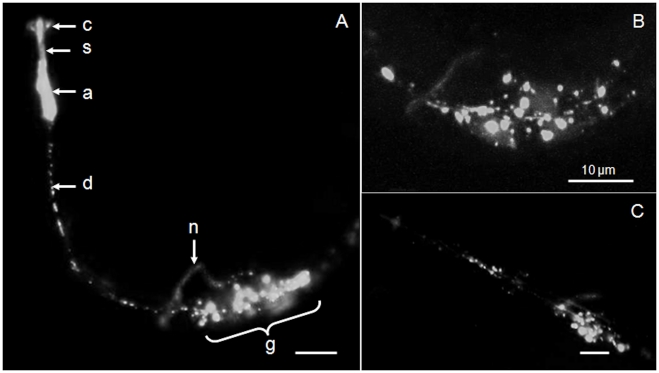
Neuronal uptake of Alexa Fluor 488-labelled nAChRbp by *Heterodera schachtii.* A) After 16 hours incubation in 183 µM peptide-dye conjugate the peptide was detected in J2 of *H. schachtii* by epifluorescence as in Figure 1. B) After a similar incubation in labelled peptide the region of neuronal cell bodies posterior to the nerve ring was observed using confocal microscopy. C) Epifluorescent detection of the labelled peptide 48 h after removal of the *H. schachtii* from the peptide solution. All images are lateral views of the nematodes. Key: c, cephalic framework; s, stomatostylet (both showing autofluorescence); a, amphidial pouch; d, tract of amphidial dendrites; n, nerve ring; g, region of the lateral and ventral ganglia. The scale bar is 10 µm and the dorsal side is that nearer to the scale bar.

**Figure 4:**
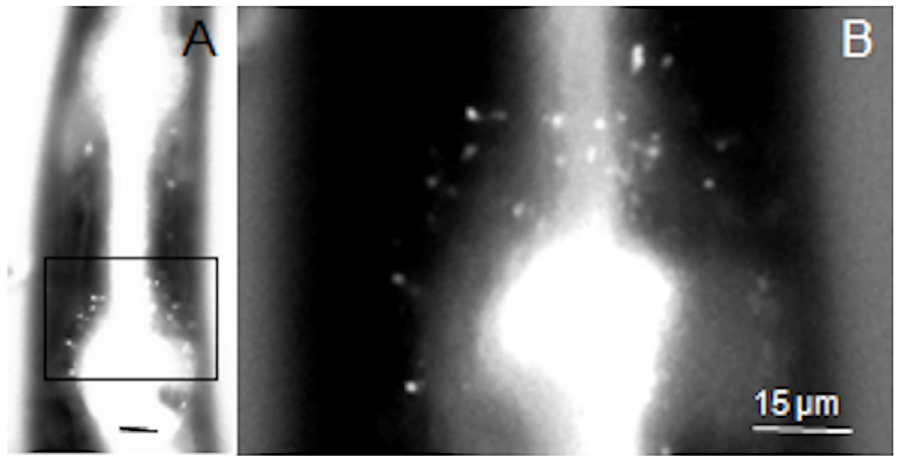
Neuronal uptake of Alexa Fluor 488-labelled nAChRbp by a hermaphrodite of *Caenorhabditis elegans.* After 18 h incubation in 300 µM peptide-dye conjugate the peptide was detected by epifluoresence as in Figure 1. A) posterior region of the pharynx and B) detail of boxed area showing fluorescence in the region of the lateral ganglia where the dye-filling neuronal cell bodies of this nematode occur. In contrast to the tylenchids this nematode ingests the fluorescent peptide causing intense fluorescence in the lumen of the pharynx. The scale bar is 15 µm; nematode anterior uppermost.

**Figure 5:**
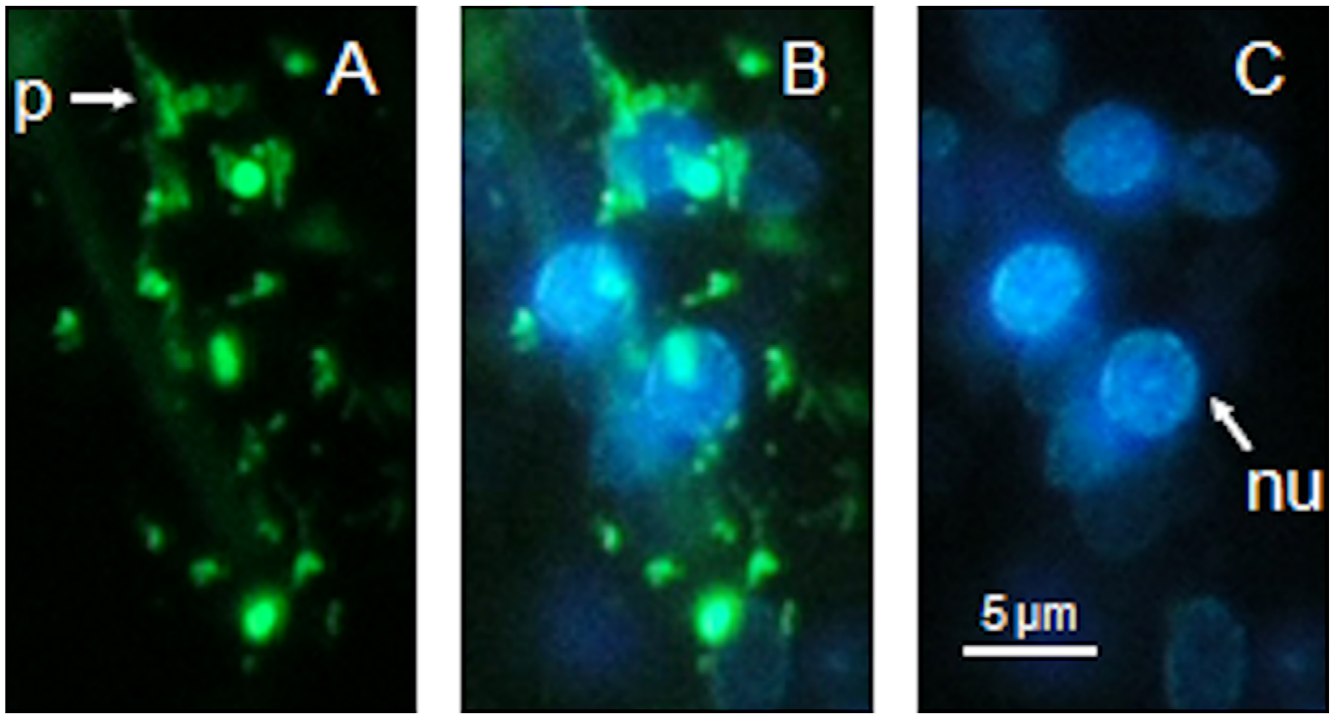
Simultaneous uptake of Alexa Fluor 488-labelled nAChRbp and bisbenzimide by a J2 of *Heterodera schachtii.* J2 of *H. schachtii* were incubated for 16 h in 183 µM nAChRbp labelled with Alexa Fluor 488 together with 1 mM bisbenzimide. The fluorophores were visualised by different excitation and emission wavelengths. Images correspond to a region 5–30 µm posterior to the nerve ring. A) Green fluorescence of the labelled peptide. C) Blue fluorescence of bisbenzimide. B) Combined image of A) and C). Key: p, process of a neuron; nu, nucleus. The scale bar is 5 µm and the images are left lateral views with the anterior uppermost.

Uptake of nAChRbp was visualized after conjugating it to Alexa Fluor 488. J2 of *H. schachtii* were placed in 183 µM of this fluorescent version of the peptide. The fluorescence detected was more extensive than for the fluorophore alone and at least as extensive as that for FITC. All nematodes examined revealed similar patterns of fluorescence. The region of the amphidial pouch was highly fluorescent for the nAChRbp/Alex Fluor 488 conjugate and it was present locally along the route of the dendrites of the amphidial neurons and also in the region just behind the weakly positive nerve ring to about 40 µm posterior to it (Fig. 3a). A key difference between uptake of labelled peptide and FITC dye staining was the aggregation of the peptide behind the nerve ring. Confocal microscopy confirmed the discrete nature of the fluorescence emissions (Fig. 3b). Fluorescence was lost from the amphidial pouches 48 h after removal of nematodes from the peptide solution but it remained extensive in the region of the amphidial cell bodies (Fig. 3c). The hermaphrodite of *C. elegans* also took up the peptide to the region of its lateral ganglia (Fig. 4) but to a lesser extent than occurred for *H*. *schachtii*. Uptake in the region of the neuronal cell bodies was studied further by placing J2 *H. schachtii* in a solution containing both 183 µM labelled peptide and 1 mM bisbenzimide. The region 5–30 µm posterior to the nerve ring showed two green fluorescent features. There was a reticulate pattern that is probably formed by neuronal processes passing among cells and intensively stained spheroids of 1.0–1.5 µm in diameter (Fig. 5a). Merging of this image with that for emission from bisbenzimide (Fig. 5c) established that the spheroids are closely associated with, but not integral to the nuclei (Fig. 5b).

**Figure 6:**
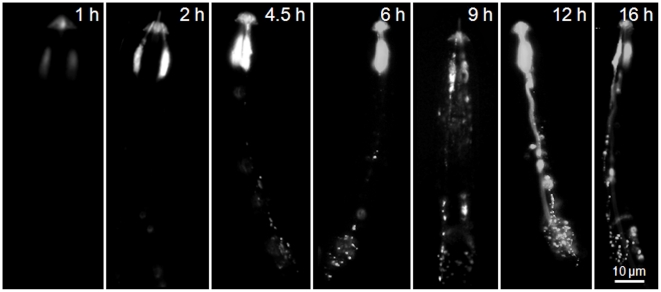
Neuronal uptake of Alexa Fluor 488-labelled nAChRbp by J2s of *Globodera pallida.* J2 of *G. pallida* were incubated in 281 µM nAChRbp labelled with Alexa Fluor 488 for varying periods of 1–16 h and the labelled peptide was then visualised by epifluorescence. Images with one amphidial pouch visualised are lateral views and those with two amphidial pouches visualised are dorsal or ventral views. The scale bar of 10 µm applies to all images.

**Figure 7:**
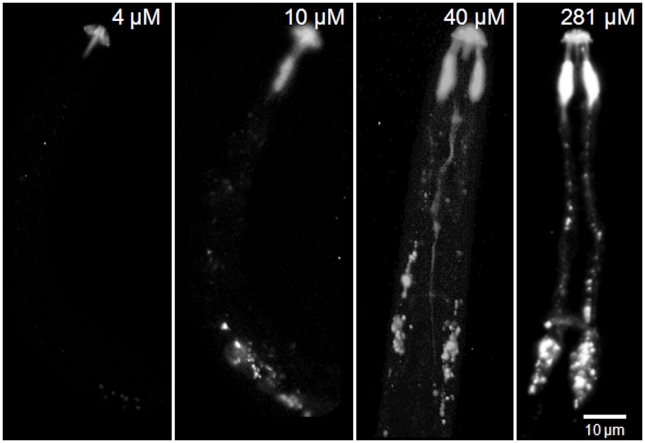
Neuronal uptake of four different concentrations of labelled nAChRbp by J2s of *Globodera pallida.* J2 of *G. pallida* were incubated for 16 h in 4 µM, 10 µM, 40 µM or 281 µM nAChRbp labelled with Alexa Fluor 488. The peptide was visualised by epifluorescence. The scale bar of 10 µm applies to all images.

The J2 of the potato cyst nematode, *G. pallida*, also demonstrated uptake of the labelled peptide along their amphidial neurons and showed a very similar pattern of fluorescence to that of J2 *H. schachtii* after 16 h incubation in 281 µM labelled nAChRbp (Fig. 6). For this nematode a time course of incubation in 281 µM labelled peptide was carried out to explore the rate of peptide transport. The labelled peptide provided a high level of fluorescence in the region of the amphidial pouches by 2 h with some fluorescence apparent in the region of the cell bodies of the amphidial neurons by 4.5 h. Emissions along the dendrite were very evident at 12 h and 16 h with some fluorescence detectable from the nerve ring at 9 h and certainly by 16 h. Two main points are apparent. After 2 h the region of the amphidial pouches had become fluorescent but the labelled peptide took about another 2 h to travel the 55–70 µm along dendrites to the region of the amphidial cell bodies. Additionally, the labelled peptide accumulated in this region with more than 4.5 h exposure to 281 µM peptide. When only 4 µM labelled peptide was used, fluorescence was not evident in the amphidial pouches, their dendrites or cell bodies at but it was visualised at both 10 µM and 40 µM with considerable uptake evident for nematodes soaked in 281 µM peptide (Fig. 7).

### Behavioural Assays

The work progressed to behavioural assays. J2s of *G. pallida* were soaked in unlabelled nAChRbp concentrations of 0, 10, 40 or 281 µM for 16 h. Individuals were then placed on agar plates at 3.5 cm from a 2 µl dried droplet of potato root diffusate and their movement detected from the tracks they left in the agar over the following 3 h. The directions of tracks were summarised in three categories. Nematodes that repeatedly moved to within 0.5 cm of the attractant were scored separately from those that did this once with the third category being those that did not approach the root diffusate that closely. Only 10 of 56 control nematodes moved repeatedly to within 0.5 cm of the root exudates. This low rate of attraction is consistent with the need to stimulate J2 of a cyst nematode with root exudates and pre-select those responding individuals before a much higher success rate is achieved [4]. This was not attempted as any pre-selection may have biased the results. Movement into the central zone of the agar plate was not significantly affected by exposure for 16 h to 10 µM peptide (Table 1). Exposure to 40 µM resulted in no nematodes entering the central zone repeatedly but a few did so once and the combined value for both these responses was not significantly less than that for the control. A concentration of 281 µM suppressed movement into the attraction zone both repeatedly and on single occasions. That effect was lost when nematodes were placed in this high concentration of peptide for the shorter period of 4.5 h. These results are consistent with more than 4.5 h being required for the peptide to accumulate to behaviour-disrupting levels in the region of the amphidial cell bodies. The concentration dependence is also evident in both the behavioural response and peptide accumulation at 16 h exposure to 281 µM rather than 40 µM peptide (Fig. 7). Furthermore, nAChRbp secreted in root exudates by transgenic potato plants also reduced attraction of J2 *G. pallida* in the assay.

**Table 1:** Effect of nAChRbp on the ability J2 *Globodera pallida* to respond chemotactically to root exudate.

Concentration of nAChRbp (µM)	Period of soaking (h)	J2 attracted to exudate			P value of χ^2^ for null hypothesis from the control	
		repeated	once	no attraction	repeated	sum of repeated and once
control (0)	16	10	0	46		
10	16	5	2	53	0.15	0.36
40	16	0	4	56	0.001	0.067
281	16	0	3	57	0.001	0.029
281	4.5	4	0	56	0.067	0.067
root exudate from transgenic plants	16	1	1	54	0.005	0.015

Nematodes were exposed to three concentrations of the peptide or to root exudate from transgenic potato plants secreting the peptide. Their subsequent movement on agar plates into a central zone surrounding non-transgenic potato root exudate was monitored over a 3 h period. Nematodes were scored into three groups from the tracks on agar relative to root exudate placed at the centre of the Petri dish. Some moved repeatedly to within 0.5 cm of the dried exudate, others did so once while many never approached the centre of the plate. The χ^2^ values were calculated after correction for continuity.

## Discussion

The amphidial neurons in *C. elegans* include some that have a pair of primary cilia forming two sensilla (ADF and ADL) or one sensillum each (ASH, ASI, ASJ, ASK). Of these ASE, ASI and ASK are involved in chemoattraction and ADL in chemorepulsion [15]. The sensilla of six amphidial neurons, ADF, ADL, ASH, ASJ, ASI and ASK dye fill [11]. The latter two are involved in chemoattraction but ASE is primarily involved in detection of water soluble molecules and it does not dye-fill [15]. The cell bodies of the dye filling amphidial neurons of cyst nematodes are grouped in a similar position to those of *C. elegans* [2] (Fig 1c) which is consistent with the evidence of some homology of these neurons in distinct nematodes [13]. The dye-filling amphidial neurons of *C. elegans* are not cholinergic. If the dye-filling neurons of cyst nematodes are also not cholinergic it is not immediately apparent how a peptide that binds to nAChRs is able to disrupt orientation to a chemical attractant. The evidence provided here provides insight into a likely mechanism.

Disruption of chemoreception probably does not occur at the sensillum because the peptide is abundantly present in the amphidial pouches within 2 h of exposure to the nematode (Fig 6) but it takes >4.5 h before orientation is prevented (Table 1). In addition nAChR receptors seem to be absent at these sensilla, although a cholinesterase is expressed in the inner labial papillae [14] and the DEG-3/DES-2 nAChR of *C. elegans* is localized to non-synaptic regions, including the sensory endings of IL2 and FLP sensory neurons [16]. Once the peptide has entered the sensilla of dye-filling neurons it probably undergoes retrograde transport from the tips of the cilia back to the transition zone at their base [17]. The dynein responsible for this (CHE-3) unloads cargo at the base of the cilium where it joins the dendrite [18] but does not participate in dendritic retrograde transport [19] and so the peptide must be re-loaded onto the retrograde transport system along the dendrite. This movement is probably similar to that in axonal transport systems of which at least three mechanisms occur. One is a fast component delivering mainly membrane-bound organelles at a rate of 0.58–4.63 µm sec^−1^ and is very much faster than occurred for the peptide in the current work. Two slow axon transport systems have been characterised, involving distinct subcomponents (SCa and SCb). SCa transports microtubules and neurofilaments at mean rates of 0.01 µm sec^−1^ in an anteriograde direction. SCb moves cargoes at somewhat quicker mean rates of 0.02–0.09 µm sec^−1^ and transports as many as 200 different proteins [20]. The approximate rates of transport in the current work can be inferred. There was no fluorescence evident in the neurons at 2 h but by 4.5 h the most advanced fluorescence in the dendrite in Fig 6 was 58 µm behind the amphidial pouch suggesting a minimum rate of transport of 0.0064 µm sec^−1^. This rate is somewhat slower than those in a retrograde direction of about 1 µm sec^−1^ in cultured hippocampal neurons of the mouse [21] and dendrites of *C. elegans* [19]. Other slow transport systems occur and their rate of movement depends on the neuron, the cargo and other factors [22]. SCa based transport is slow partly because movement is not continuous. A short mobile phase and a long, intermittent stationary phase that may represent 97% of the total journey time are iterated as the neurofilament is transported [23].

Once the peptide reaches the proximal end of the dendrite it must be transported to a cholinergic synapse with nAChR receptors for an effect to occur. Its apparent localisation in the perinuclear region of neuronal cell bodies suggests it may become associated with the *trans*-Golgi network (Fig 5). It is here that neuropeptide precursors and accessory enzymes required for their processing are organised into dense vesicles for fast transport along the axon [24] and exocytosis at the pre-synaptic membrane. It is therefore possible that the peptide could be transmitted by this mechanism. Once released into the synaptic cleft, the peptide would be able to bind to any nAChR receptors on the post-synaptic membrane of a neuron. Fluorescence in the nerve ring shows the peptide is present where interneurons and amphidial neurons connect (Fig. 3a,b). This is of interest because the neurotransmitter acetylcholine occurs in the nerve ring of *C. elegans.* It is synthesized by choline acetyltransferase and loaded into synaptic vesicles by a specific vesicular acetylcholine transporter. Both proteins are abundantly expressed in the nerve ring [25]. Therefore the nerve ring is a site of nAChRs that the peptide does access after uptake by the dye-filling neurons. The peptide is also present immediately posterior to the nerve ring and in the region of the lateral ganglia of *C. elegans* (Figure 4) where the cell bodies of both the dye-filling neurons and the interneurons to which they connect occur.

The interneuron AIA is of particular interest assuming functional homology of the chemoreception pathway of cyst nematodes and *C. elegans*. It is a primary interneuron that is cholinergic and receives input from sensory neurons including ASI which dye-fills. Interestingly, the axon of AIA connects to AIB and these two interneurons play a central integrative role in chemotactic behaviour. They both may regulate turning [26] with AIA showing time-varying acute responses and AIB sustained responses [27]. Ablation of AIA does not prevent chemotaxis but it may influence pirouette frequencies [28]. The inability of *G. pallida* to orientate towards root diffusates is consistent with a role for the peptide in disrupting the orthologues of AIA and hence AIB function in the cyst nematode. Unfortunately the peptide was taken up to a much lesser extent by *C. elegans* than by the tylenchid nematodes (cf. Figures 3 and 4) and effects on its pirouetting behaviour were not detected (data not presented). The lack of any reduction in speed of locomotion of cyst nematodes suggests that the command neurons are unaffected by the peptide uptake. We conclude that orientation of *G. pallida* to host roots is likely to be lost when the peptide is taken up by a limited number of chemoreceptive neurons and delivered by them to post-synaptic junctions of cholinergic neurons possibly including the homologue of the AIA interneuron of *C. elegans.* The combination of a non-lethal mode of action, distinctive uptake pathway and inferred targeting to cholinergic neurons in the chemoreceptive pathway suggest non-target effects should be limited. Even non-target nematodes that take up the peptide as efficiently as cyst nematodes will experience a transitory effect if they move from close proximity to the root.

## Materials and Methods

### Nematode hatching

Second stage juveniles (J2s) of *H. schachtii* and *G. pallida* were hatched from cysts soaked, respectively, in 3 mM ZnCl_2_ or potato root diffusate as previously described [29].

### Fluorescent dye-uptake

The FITC staining protocol was modified from a standard method [10]. A 50 mM stock solution of FITC in dimethyl formamide was diluted to 1 mM in distilled water immediately prior to use. Second stage juveniles (J2s) of *H. schachtii* were soaked in the FITC solution at room temperature in the dark for 16–24 h with approximately 100 nematodes in each 0.5 ml tube. Alexa Fluor 488 dye (Invitrogen, Paisley UK) was dissolved directly in distilled water at a concentration of 1 mM. J2s of *H. schachtii* and *G. pallida* were soaked separately in 20 µl of the dye solution in the dark at room temperature for 16–24 h.

### Neuronal uptake of fluorescent peptide

The nAChRbp was synthesized and then labelled by the fluorescent dye Alexa Fluor 488 at its carboxyl end. The labelled peptide was cyclized and purified to >95% by high performance liquid chromatography (HPLC) analysis and free dye eliminated. The above procedures were carried out by a specialist commercial company (Cambridge Research Biochemicals, Cambridge, UK). J2s of *H. schachtii* were soaked in a 183 µM aqueous solution of the Alexa Fluor 488-labelled peptide at room temperature in the dark for 16 h. The stability of the tagged peptide within nematodes was studied by incubating J2s of *H. schachtii* for 16 h before washing them with several changes of water over 48 hours. Experiments with *G. pallida* J2 were conducted in a similar manner. In this case incubations were carried out either in a range of peptide concentrations (281 µM, 40 µM, 10 µM and 4 µM) for 16 h or at a concentration of 281 µM for varying time periods of 1, 2, 4.5, 6, 9, 12 and 16 h. Hermaphrodites of *C. elegans* were incubated for 18 h in 300 µM labelled peptide in M9 buffer.

For simultaneous neuronal uptake of fluorescent peptide and bisbenzimide, J2s of *H. schachtii* were soaked in a distilled water solution with 183 µM Alexa Fluor 488 labelled nAChRbp plus 1 mM bisbenzimide at room temperature in the dark for 16 h.

### Behavioural assays using J2 of *G. pallida*

Potato root exudate for use as an attractant in chemotaxis assays was obtained from tissue culture plantlets of potato cv. Désirée. Shoots cultured on solid medium were rooted in liquid MS medium supplemented with 0.1 mg l^−1^ NAA. After 25 days growth, when strong root systems had developed, plantlets were transferred individually to 50 ml tubes containing 7 ml sterile tap water. They were kept in a growth chamber for 20 days during which time the volume of water in each tube was maintained at 7 ml. The water was then collected and used as potato root exudate. Root exudate was collected from potato cv. Désirée transformed with a construct to allow secretion of the nAChRbp [10] in a similar manner. A radial concentration gradient was established by applying 2 µl of the root exudates from untransformed potato plants to the centre of a 9 cm Petri dish containing 8 ml of agar medium (2% Micro Agar (Duchefa Biochemie), 0.25% Tween 20, 10 mM Hepes at pH 7.2) [30]. The plates were left at room temperature for 12–16 h before use as described [30]. Newly hatched J2s of *G. pallida* were divided into six groups of about sixty nematodes each. Five groups were soaked in either a) 20 µl tap water, b) potato exudates containing secreted peptide, c) 10 µM d) 40 µM or e) 281 µM Alexa Fluor 488 tagged nAChRbp in distilled water at room temperature for 16 h (Table 1). The sixth group was soaked in 20 µl of the high concentration of 281 µM tagged peptide but for 4.5 h only. Each group was rinsed once in 200 µl tap water for 5 min before being put onto plates. Two J2s of *G. pallida* were placed on opposite sides of the agar plate at 1 cm from the periphery. They were allowed to orientate at room temperature in the dark for 3 h before those individuals that had migrated into a 0.5 cm radius circle at the centre of each plate were determined from tracks left in the agar.

### Behavioural assays using *C. elegans* adults

At the first day of adulthood *C. elegans* were incubated for 18 h in M9 buffer or M9 buffer containing 300 µM unlabelled form of the peptide in M9 buffer. Individuals were transferred to 6 cm agar plates containing *E. Coli* OP50 for 24 h prior to transferral to separate agar plates without food for monitoring of pirouetting behaviour [31].

### Fluorescent microscopy

After the required incubation period, dye or tagged peptide solution was removed and nematodes were washed 3–5 times with distilled water. For observation, batches of 5–10 animals were picked out with a fine needle and mounted on a 2% agarose slide containing 2–10 µl 0.01 M sodium azide to immobilize them.

Bright-field images were observed using a compound microscope (Leitz DMRB) at 400x magnification. Green fluorescence filter sets (Leitz) with excitation wavelength of 480±20 nm and emission wavelength of 510 nm were used with the same microscope for visualisation of FITC and Alexa Fluor 488. Violet fluorescence filter sets (Leitz) with excitation wavelength of 425±20 nm and emission wavelength of greater than 475 nm were used for visualisation of bisbenzimide. Images were captured with an Olympus C-5050, except those of *C. elegans* for which a QImaging QICAM Monochrome Camera was used and Media Cybernetics Image-Pro analyser 7.0. Confocal images were obtained with a Zeiss LSM 510 META Axioplan laser scanning microscope using an Argon 488 laser line.
